# The ethics of research in compulsory drug detention centres in Asia

**DOI:** 10.7448/IAS.15.2.18491

**Published:** 2012-12-05

**Authors:** Richard Pearshouse, Joseph J Amon

**Affiliations:** Human Rights Watch, New York, NY, USA

Throughout China and southeast Asia, people who use drugs are compulsorily detained in government centres in the name of “treatment” or “rehabilitation”. These centres are neither prisons nor hospitals: individuals are held without the due process protections common to prisons, such as access to legal counsel, the opportunity to appeal, or judicial oversight of detention. At the same time, they may be detained for years without ever receiving evidence-based drug dependency treatment. Detainees may be held in isolation cells, forced to work, and ill-treated by staff. In some centres, detainees may be tortured with electrical shocks or sexually abused. Individuals are detained without clinical determination of drug dependence, and centres also lock up homeless people, street children, and people with mental illnesses [1–5].

Individuals in these centres are also routinely denied basic health care. Yet detainees may come in contact with health professionals: they may be subject to mandatory HIV testing [2], forced to donate blood [4], or they may participate in scientific research, sometimes involuntarily [6]. Published research in drug detention centres has investigated issues such as risk factors for HIV infection among injecting drug users (IDU) [7–10], HIV prevalence [11], effectiveness of behavioural HIV/STI prevention interventions [12], and the efficacy of particular modalities of drug treatment [13,14].

While involuntary research is clearly unethical, there has been little discussion of the ethical considerations of research on individuals in extra-judicial compulsory drug detention centres. A fundamental requirement for scientists conducting such research should be to accurately describe the status of research participants and the research setting. However, published research frequently omits mention of the involuntary detention of individuals [11,14], or does not describe the length of their detention or the non-availability of evidence-based treatment [7,8]. Research has referred vaguely to the “complex” legal needs of detainees [12], or mischaracterised their legal status by describing detainees as “in-patients” [14] or “residents” (with those detained by police being “police-referred”) [7].

Hien and colleagues use several euphemisms to describe the experiences of detainees in one centre in Ho Chi Minh City, saying that it provides “physical exercise, education, information, and job training for drug users in the city” [9]. By contrast, our research conducted with former detainees of the same centre, found that they are forced to work without pay for up to eight hours a day manufacturing garments. “Labour therapy” performed on a compulsory basis is a legally mandated component of drug treatment in Vietnam (and was at the time of the study) [3].

Accurately identifying the setting also allows readers to assess the ethical adequacy of study design. For example, ensuring valid informed consent in coercive settings is not straightforward, yet some research reports do not mention whether they sought the informed consent of participants [8], or state (without further elaboration) that informed consent was obtained when “all available and eligible residents [of one centre] were invited and accepted study participation” [7]. Hien and colleagues note that the study refusal rate among detainees was considerably lower than the refusal rate among drug users on the street. They speculate that “street IDUs may not have understood the purpose of the study, leading to refusal to participate”, without considering alternative explanations (including coerced participation of detainees) [9]. Shen and colleagues ignore the potential of coercion when they claim that they ensured valid informed consent from participants by having detention centre staff witness that consent [12].

Research in any setting should be conducted with due regard for the ethical principles of respect for persons, beneficence, and justice; ethical guidelines impose strict standards for research involving prisoners [15–17]. However, drug detention centre detainees are not legally prisoners and in many respects are more vulnerable. Detainees cannot seek legal counsel in the case of unethical research or recriminations from declining to participate or seeking to end participation in the study. Human rights monitors are not allowed to enter and speak privately to detainees. The absence of independent judicial oversight and the lack of transparency and accountability in the management of these drug detention centres make it difficult, if not impossible, for researchers, or prison research subject advocates, to monitor and respond to potential negative consequences from research participation.

Research in drug detention centres that does not reflect upon the conditions in the centre or the legal status of detainees does tangible harm to detainees: it disguises recognition of these centres as settings of gross human rights abuses, and may be used by governments to claim these centres as legitimate institutions [18–20].

When challenged on the ethics of their research, some researchers have defended their research by stating that they did not identify abuses in the centres [21]. Such a rebuttal is unsurprising when researchers do not gather data on human rights abuses [14], or are unfamiliar with legal definitions of forced labour or other abuses [21]. Bioethicists have also defended research in detention centres, without apparent understanding of the legal context or conditions facing detainees [22].

It is not accidental that studies conducted in drug detention centres fail to expose the real nature of such centres and are silent on the conditions experienced by those inside. Human rights monitoring by independent international organisations is not allowed in many countries that detain drug users in “re-education” centres, and access to these centres is strictly controlled. For researchers, the bottom line may be, as Thao and colleagues acknowledge, that detained drug users are an “easier” population to recruit than street-based subjects [10].

Researchers should not carry out research in drug detention centres that can be conducted in the community, unless there are ethically sound reasons for doing so. Journal editors should scrutinise research conducted in compulsory “treatment” centres, and reject research that does not clearly identify adequate ethical protection provided to research participants and describe the context and conditions of the research setting.
